# Enhancing access to primary care is critical to the future of an equitable health service: using process visualisation to understand the impact of national policy in the UK

**DOI:** 10.3389/frhs.2024.1499847

**Published:** 2025-01-27

**Authors:** Ian Litchfield, Nicola Kay Gale, Sheila Greenfield, David Shukla, Micheal Burrows

**Affiliations:** ^1^ Department of Applied Health Sciences, College of Medicine and Health, University of Birmingham, Birmingham, United Kingdom; ^2^ Health Services Management Centre, College of Social Sciences, University of Birmingham, Birmingham, United Kingdom; ^3^ Eve Hill Surgery, Dudley, United Kingdom; ^4^ School of Psychology, University of Coventry, Coventry, United Kingdom

**Keywords:** primary care, policy implementation and delivery, access and quality, process visualization, National Health Service England

## Abstract

Access to UK general practice is complicated by the need to provide equitable and universal care within a system adapting to workforce challenges, digital innovation, and unprecedented demand. Despite the importance of accessing primary care in meeting the overall aim of delivering equitable care, this is the first time the direct and indirect influence of policies intended to facilitate access have been systematically explored. Further consideration by policymakers is needed to accommodate the difference between what patients need and what patients want when accessing primary care, and the differences in their ability to utilise digital options. The designation of care was hindered by long-standing issues of reliable data and variations in the interpretation of local and national protocols and guidelines.

## 1 Introduction

Although health care systems worldwide possess different financial motivations, staffing models, clinical capability, and capacity, they are all predicated on facilitating timely and appropriate access to care (1–4). In high income countries this access tends to begin with primary care, more specifically general or family practice where it is facilitated by a range of facilities, localities, clinical disciplines, and digital components (5). However, despite the best efforts of policymakers, funders and senior decision-makers, delays and inequities in access persist across multiple health systems (6). These are exacerbated by a lack of evidence that contextualises the implementation and interaction of central health care policies with the localised influences of individual primary care organisations, health care providers, and patients (7).

In the United Kingdom (UK) issues around access to healthcare, in particular access to primary care, have risen to national prominence, garnering the interest of the mainstream media and becoming the focus of political debate (8). The Royal College of General Practitioners (RCGP), has expressed concern that there has been no systematic attempt to explore the failings and strengths in securing access to primary care (9). This includes the contextual influences of patient needs and preferences, staff attitudes, training, and experience, and the various characteristics of primary care organisations (10).

Over the last decade the National Health Service England (11) has introduced multiple policies and various funding streams in an attempt to improve primary care access; these include those directly aimed at a specific elements of access such as improving telephone systems, or increasing the use of digital first appointment booking; those more broadly aimed at the scope and delivery of primary care, such as the inclusion of additional non-clinical roles into the practice team, and finally the broader delivery of the health service such as the move to integrated health and care systems as described in Table 1. In all cases, their implementation as it affects primary care has been complicated by the growing demands of an ageing population, increasingly complex options for treatment and care, the challenges of reduced GP recruitment and retention (31, 32) and an evidence base limited in focus to discrete patient groups defined by condition or age (33–35). The work presented here uses process visualisation, namely a Service Blueprint, to unpick the process of primary care access and in turn the influences of individual and collective policies, ultimately providing a series of recommendations for future policy development applicable both to the NHSE and elsewhere.

**Table 1:** Summary of key policies in relation to primary care access.

Area of service	Author	Name	Year
System wide	Department of Health	Health and social care act (12)	2012
	NHS^a^	NHS Long term plan (13, 14)	2020
	NHS England	Integrated care systems.	2020
	NHSE^b^	Artificial intelligence (AI) and machine learning (15)	2023
	NHSE	Social prescribing (11)	2022
Primary care	Primary Care Workforce Commission	The future of primary care: Creating teams for tomorrow. (16)	2015
	NHSE	General practice forward view (17)	2016
	NHSE	Digital First Primary Care (18)	2021
	NHSE	A five-year framework for GP contract reform to implement the NHS long term plan (19)	2019
	NHS Improvement	Network Contract Directed Enhanced Service: Additional Roles Reimbursement Scheme Guidance (20).	2019
	NHSE	Self-referral for tests and appointments for hundreds of thousands of patients (21).	2024
Specific to primary care access	NHSE	NHSE 111 service (22)	2022
	NHSE	Improving access for all: reducing inequalities in access to general practice services (23)	2018
	NHSE	Using online consultations in primary care: implementation toolkit (24).	2020
	NHS Digital	Digital First online consultation and video consultation framework (25)	2022
	NHSE	Delivery plan for recovering access to primary care (26)	2023
	British Medical Association.	Care navigation and triage in general practice (27):	2023
	NHSE & NHS Improvement	Advice on how to establish a remote ‘total triage’model in general practice using online consultation. (28)	
	NHSE	Delivery plan for recovering access to primary care: update and actions for 2024/25 (29)	2024
	NHSE	How to improve telephone journeys in general practice (30)	2024

^a^ National Health Service.

^b^ National Health Service England.

### 1.1 Process visualisation

In the absence of any previous depiction of the process of accessing primary care we created a Service Blueprint (36, 37), a tool widely used in designing, delivering, or understanding new and established (health) service offerings (38–40). They have been used in a range of contexts that involve multiple people, processes, and channels of communication (40). They have been successfully applied to a range of healthcare environments and processes including the exploration of digital healthcare (41, 42), shared provider-patient decision making (43, 44), and delivering patient centred care (45, 46).

**Figure 1:**
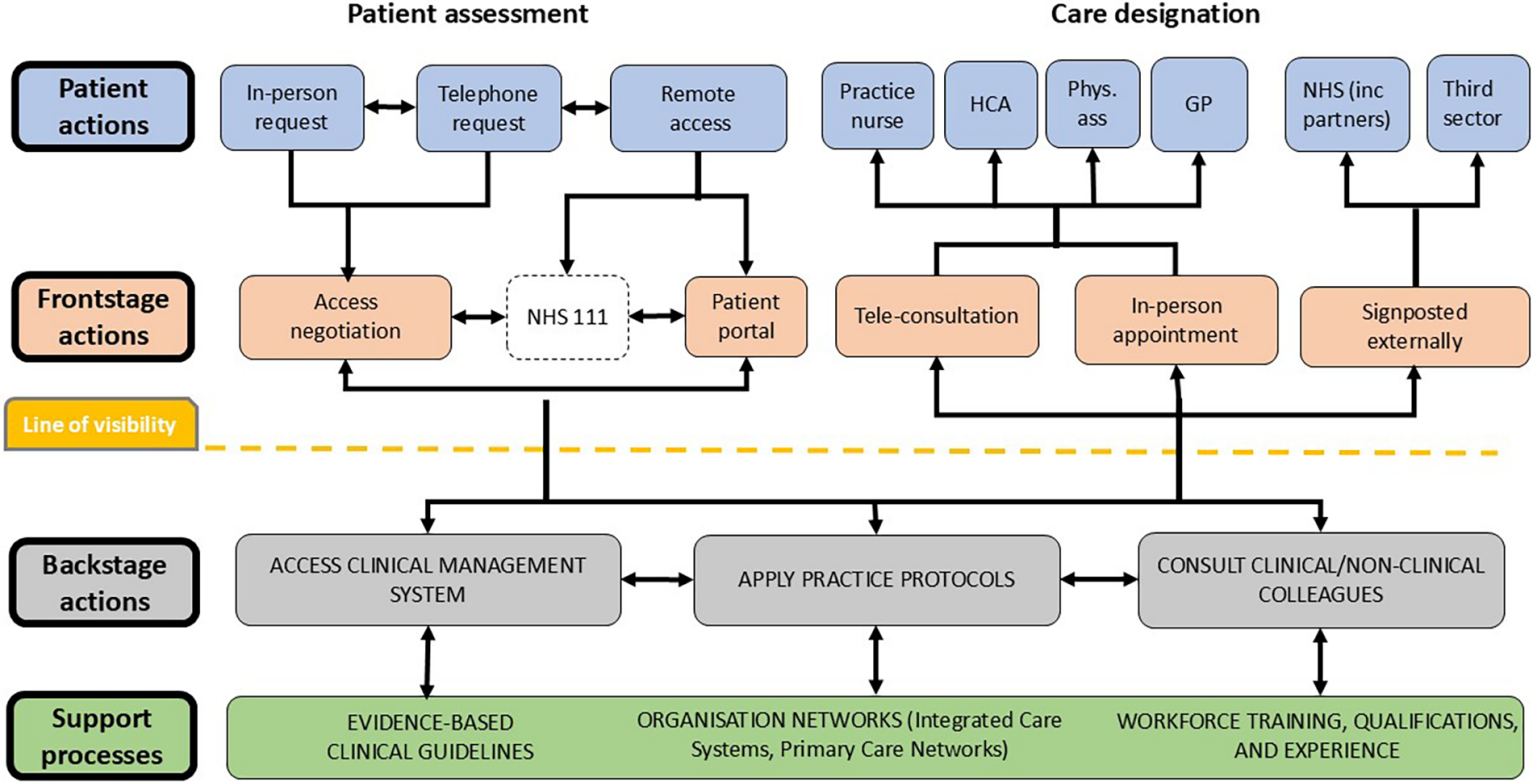
Service blueprint showing the process of access to general practice.

The service blueprint was developed using a secondary descriptive qualitative analysis relating to the procedural aspects of accessing care drawn from the lived experience of 52 staff (including GPs, nurses, practice managers, and receptionists) and 27 patients from five practices within the English Midlands (47). This was.corroborated by documental evidence drawn from practice protocols, independent reports, and existing academic literature (48). The resulting blueprint describes the individuals involved, their roles, actions, and support systems within two phases, the first is Patient Assessment, consisting of the initiation of contact by patients and the subsequent contact with service providers, including provider information gathering and patient negotiation; the second is Care Designation, describing the allocation of care whether within the practice or external settings or sources of support (49, 50). See Figure 1 for the blueprint describing access to primary care.

## 2 Policy options and implications

### 2.1 Patient assessment phase

#### 2.1.1 Patient/frontstage actions

##### 2.1.1.1 In-person/telephone request

Access typically begins with patients’ synchronous in-person or telephone contact with practice staff (51). Traditionally patients in the UK would be expected to access practices as a walk-in patient reflecting the history of general practice where single-handed practices serving smaller numbers of patients were the norm (52). In the last two decades the growth in size, and reduction in number of practices has meant their boundaries have been extended and such walk-in appointments are becoming rarer, with a marked increase in telephone contact (53). However, this is not universal and, in areas of high-deprivation, frustration with remote access and in some cases individual practice organisation protocols, means that attending in-person remains a preferred, or only viable option (54).

Currently patient attempts to access general practice in NHSE begin with contacting the practice via telephone (51). The ease of telephone access has been under closer scrutiny since the pandemic when what were already sometimes significant waits have become significantly longer (55–57). The additional burden of these waits can discourage some from seeking care entirely (57–61). In response in 2023 NHSE introduced ring-fenced funding to improve call management systems though their impact is as yet unexplored (62).

Patients can also contact NHS urgent care without needing a GP referral via the NHS 111 clinical assessment service system (63), a service designed to provide advice and signposting for people with urgent health-care problems. However, latest evidence suggests it has done little to reduce the pressure on direct contact with practices as it is seldom used by older or less well-educated patients i.e., the population groups most frequently in contact with general practice (64–66).

##### 2.1.1.2 Online access

The NHSE have attempted to relieve some of the acknowledged pressure on front-line administrators by providing patients with the opportunity to independently book routine appointments on-line via patient portals and the multi-purpose NHS App (67, 68). In an attempt to broaden its use NHSE have issued detailed guidance to help practices with their implementation and integration of online booking (24). The latest iterations of these booking portals are growing in sophistication to include online symptom checkers, mechanisms for self-referral, and closer interaction with the NHS 111 system (63, 66, 69–71). All are available through the NHS App (68).

Take up of online booking is growing more slowly than anticipated, and used disproportionately by those that are younger and more affluent (57, 70, 72, 73), highlighting the discrepancies between UK policymakers move to digitalisation and the reality of it exacerbating existing health inequalities (74).

The use of online portals and their associated symptom checkers offers the potential of a streamlined automated booking service that can use algorithms to direct patients to the most appropriate care, but to function as expected they must be fed reliable data which currently does not exist, a precept for the expected use of AI in the role (26). Currently the efficacy of these systems means that the benefit of longitudinal contextual understanding of individual patients is lost, alongside that of in-person contact when assessing patients’ clinical need (75–79).

##### 2.1.1.3 Access negotiation

For patients contacting the practice in person, the member of the practice team that typically processes patient requests is the receptionist (51), a role requiring no formal training or qualifications (75). In performing this role, receptionists assimilate formally described medical data from the patient's electronic health record (EHR), GP notes and recommendations, alongside patient descriptions of their symptoms and other contextual information, though not all patients are willing to disclose medical information to non- medically trained receptionists (51, 80).

Subsequent negotiations can be complicated, not every patient wants the same outcome, for example some might want the earliest available appointment but others a consultation with their usual or preferred clinician (51). It is also important that socio-cultural influences are accommodated, for example those from black and minority ethnic backgrounds prefer to wait for a clinician of the same gender (23, 81, 82) or the elderly may prefer in person appointments instead of teleconsultations (55).

This highlights the importance of understanding the discrepancy between what patients want from that initial contact and what commissioners and policymakers consider a successful outcome (83). Though waiting times for GP appointments are seen as the key metric, meeting patients’ expectations for seeing their preferred GP is currently not routinely recorded (84–87).

### 2.2 Care designation phase

#### 2.2.1 Patient/frontstage actions

Various options are available when allocating care for patients including in-person, telephone or virtual appointments. Though traditionally this appointment would be with a GP they are increasingly conducted with an alternative member of the practice's clinical team or signposted to a source of care and support external to the practice (27, 88).

##### 2.2.1.1 Teleconsultations

In 2019 NHSE declared that all GP practices should promote and offer remote i.e., telephone, online, or video consultations to their patients as an option for consulting with busy clinicians (19). This Digital First model offers patients a face-to-face consultation only when deemed necessary (26, 89, 90). Since COVID, teleconsultations have become the most frequently offered option in UK general practice (91). The use of digital routes is intended to make access easier; however, failure to account for the lack of connectivity and digital literacy in underserved populations is leading to an exacerbation of health disparities through the “digital divide” (92).

##### 2.2.1.2 In-person practice appointments

Decisions on directing patients to in-person practice appointments are informed by capacity and clinical need, including whether they should be seen by the GP. However, recent NHSE initiatives have intended to ease the burden on busy GPs by filtering patients towards alternative members of a more multi-disciplinary practice team (67, 93–96). To further support this the NHS introduced the Additional Roles Reimbursement Scheme, consisting of 17 new roles intending to improve access for patients. This includes clinical roles such as Advanced Nurse Practitioners (97), and practice-based physiotherapists (98), or pharmacists (67, 99, 100) as well as new non-clinical roles such as social prescribers (101) and health and well-being coaches (102). It is understood that for these to become an accepted option patients need education as to their value and role in supporting health (9). Physician Associates are being increasingly used in general practice, although they remain controversial with the British Medical Association asserting that they pose a risk to patient safety due to their lack of clinical training (103). The most recent evidence suggests that these initiatives have broadened expertise but failed to reduce GP burden, with issues around the scope and expectations of those in additional roles, their management and support infrastructure, and ultimately their sustained integration and career progression (104).

##### 2.2.1.3 Signposted externally

In recognition that people's health and wellbeing are determined predominantly by a range of social, economic and environmental factors, the NHS Long-Term Plan includes a commitment to offer more effective navigation across the health and social care system in an attempt to address these social determinants of health (67, 105, 106). The NHS is also placing a growing emphasis on the use of third sector organisations (e.g., charities, social enterprises and community groups) (107), facilitated by the growing role of social prescribers (108) and other lay health workers who provide synthetic forms of social support in the community (109). These individuals have basic training and are responsible for referring patients to a range of public, voluntary and community sector organisations with a focus on improving their health and well-being (108, 110). Despite their proliferation there is little evidence of their efficacy (111).

Patients might be signposted to community-based resources associated with, or contracted by, the NHS including local pharmacies (112). These have long been considered a useful opportunity to relieve the pressure on access to primary care and in 2023 the Department of Health's policy directive Recovering Access to Primary Care included funding and training to support patients attending pharmacies for the prescription of antibiotics and a range of other care solutions (62, 113–115).

### 2.3 Patient assessment and care designation

#### 2.3.1 Backstage actions

These ‘backstage’ actions performed by front line service providers happen beyond the eye of the patient and include staff accessing clinical management systems, consultation with colleagues, and referral to practice protocols.

#### 2.3.2 The clinical management system

In designating care, staff are required to locate the relevant patient information from within their practice's software based clinical management system (CMS) which incorporates the electronic health record, booking facilities, notes from GPs, reminders, referral letters and other patient-related information (116, 117). The patient data stored on the CMS is fragmented and not always current with longstanding issues around governance and interoperability with similar clinical systems in secondary care (118–121). Though a widely acknowledged concern, progress on linking data sets across NHS settings has been slow (67, 122, 123), despite NHS Supply Chain publishing open standards that technology suppliers must now comply with (67, 124).

#### 2.3.3 Consulting with practice colleagues

The BMA has recently released guidance on triage and signposting in general practice recognising the previous absence of formal recommendations (27). Prior to this general practice receptionists typically relied on formal and informal advice, and shared responsibility and accountability with clinical and non-clinical members of the practice team (93, 125). The level and consistency of the support they receive from their colleagues is dependent upon the culture of individual practice organisations and the experience of those approached (126).

The success of such inter-professional connection relies on non-judgmental channels of communication and a working culture that flattens organisational hierarchies (127–129). With the scope and scale of primary care organisations expanding, informal and formal communication has been supported by the successful introduction of micro-teams consisting of GP, administrator, and a nurse or health care assistant (93, 130–132).

#### 2.3.4 Reference to practice protocols

In the UK individual general practices develop their own protocols to support patient access, often informed by the interpretation and implementation of national and local policies and priorities (27, 133). Although there is some lattitude the overall performance of practices is regulated by the Clinical Care Commission (134). The protocols relating to access can incorporate a number of different elements according to whether usual care or urgent cases and the criteria for same-day access (35, 54, 135).

Adherence to these protocols varies some of which is due to vague definitions and poor understanding of protocols and processes (86, 87, 136, 137). Some of the variation is due to the discretion of individual staff members, a discretion given implicit legitimacy by senior practice colleagues who acknowledge that receptionists must be flexible in their approach to accommodate limited resources (54, 126). Such discretionary decision-making, and its impacts on the delivery and outcomes of broader policies has been witnessed in front line providers in other public sector services, where it has been earned the term street-level bureaucracy (126, 138, 139).

### 2.4 Support processes

The processes and policies that underlie primary care access include those that facilitate an extended primary care network, the impact of nationally implemented clinical guidelines, and the policy driven initiatives for training and qualifications of those facilitating access to care.

#### 2.4.1 Extended clinical networks

The UK Health and Social Care Act of 2022 has seen the integration of health and social care in new bodies called Integrated Care Systems (ICS) designed to unite NHS organisations, social care providers, and local authorities in planning and delivering locally relevant services (140). These are run by Integrated Care Boards with their stakeholders drawn from across care settings and communities (141). It is expected that ICSs will reinforce previously incoherent links between primary, secondary, and social care though there have been calls for the Department of Health and Social Care to remodel existing funding frameworks to incentivise greater integration and shared responsibility (87, 142).

The latest evidence suggests that primary care leaders and managers remain unclear about the role of general practice within these integrated models (143). There are also concerns that GPs’ priorities will be overshadowed by the larger funding and political influence afforded acute trusts (141). This is problematic in the context of expectations that primary care's management of chronic conditions and provision of preventative care will alleviate much of the pressure on secondary care (144, 145).

#### 2.4.2 Evidence-based clinical guidance

The delivery of evidence-based medicine in general practice is directed by national guidance intended to support equitable and consistent care (146, 147). These guidelines are expected to underpin consistent, high quality care, through their local implementation and integration with existing protocols and processes (148). They include elements of access and signposting yet these guidelines are not always followed despite the introduction of financial incentives (149). A number of reasons for this have been identified including uncertainty surrounding their relevance to patients, inadequate remuneration, or technical support, and an underlying lack of resource necessary to deliver them (150). Subsequently there have been calls for greater engagement of those creating these guidelines with representatives of the various organisational, social, cultural, and community contexts in which they will be implemented (150, 151).

#### 2.4.3 Training and qualifications for access

As described elsewhere, in UK primary care those most frequently charged with facilitating access are receptionists, unqualified but expected to fulfil a range of functions including making consequential decisions on patient priority and access, and including acting on red flags if patients present potentially serious symptoms (51, 54, 148). Recently, this aspect of their role has been acknowledged as distinct and worthy of NHSE policymakers as recategorization as care navigators and specialised training to signpost patients to various sources of help, advocacy and support (87, 96, 137, 152–154).

The growing role of remote triaging or otherwise processing patient requests remotely is made more difficult by the loss of visual cues (51, 54, 137, 155–157). Its growing prominence has been recognised as deserving of specific training both for clinical and non-clinical members of the practice team (9, 62).

## 3 Actionable recommendations

We have summarised the issues uncovered despite or because of existing policy initiatives and suggested practicable mitigations in Table 2.

**Table 2:** Actionable recommendations.

Phase	Areas of process	Task	Challenge	Mitigating solution
Patient assessment	Patient/frontstage	In-person/telephone request	Lengthy waits on busy telephone lines increasing anxiety in patients and discouraging others from seeking care	The reorganisation of telephone systems, staggering the times which emergency/same day appointments are released to avoid excessive waits whenever practice telephone lines open (typically 9 am). Include call back option (24)
				Targeted messaging and communication aimed at those groups of the population that are under-utilising or unaware of the NHS 111 system or the NHS App (152).
		Online access	The demographics of patients using online booking portals is disproportionately skewed to younger, and better educated patients.	To ensure that access remains equitable there needs to be investment in training and support for those patients not comfortable or capable of using digital services, including maintaining and enhancing other modes of access to support higher need patients (156).
		Access negotiation	There are issues in inconsistency of access through poor adherence to practice protocols by staff or otherwise the inconsistent application of discretion by those negotiating with patients.	The protocols and processes involved in access should be universally communicated to patients (and staff). To ensure more consistent conversations scripts can be provided for staff determining the flow and content of the conversation (119).
				The increased use of care navigators would offer an alternative solution that introduces more personalised care into the process at an earlier stage (94).
				Improved data collection on meeting patient preferences (158).
Care designation	Patient/frontstage	Teleconsultations	The digital first models have mandated teleconsultations. These can impact patient physician alliance and exacerbate the digital divide.	Ensure that prioritising patients for in-person appointments acknowledges that some do not have access to alternative options. Addressing technical issues at practice level can support better engagement with patients (10).
		In-person practice appointments	In-person GP appointments are becoming rarer and patients are being increasingly directed to alternative members of the practice team	Ensure that referral to alternative members of the practice team (as opposed to the GP) is safe and appropriate—also that the messaging is clear so patients understand the benefits of seeing care providers other than their GP (41).
		Signposted externally	Patients are signposted to a range of external services but with little evidence of their efficacy	Conduct audits, evaluation and research to understand whether signposting is appropriate, and patients are following recommendations. Ensure that investment in social prescribers and other lay health workers is informed by the latest evidence (71).
Patient assessment and care designation	Backstage actions	The clinical management system	Accuracy of decisions on patient access impacted by the lack of interoperability of data systems across primary and secondary care.	Cross system data linkage is not likely to be universal for some time, in its absence other measures can be taken to improve communication between settings for example ensuring that discharge letters are more accurate and delivered promptly (125).
		Consultation between practice staff.	Currently the lines of communication between staff members (seeking advice on appropriate access are informal and can lead to advice of varying relevance and quality.	It is important for practices to maintain an open, learning environment where professional hierarchies are flattened, and questions are encouraged (145).
		Reference to practice protocols	There is variation in the interpretation of practice protocols by front line staff	The influence of senior staff on adherence to patient protocols should be considered (4)
Patient assessment and care designation	Support processes	Extended clinical networks	There remains a lack of true integration across the health and social care system	The use of financial incentives to reward integration and shared responsibility (38, 138).
				Employing strategies to encourage greater integration including a common agenda; continuous communication; and shared measurement (35, 133)
		Practice protocols/Evidence based clinical guidance	Clinical guidance for access not always followed at a practice level.	Coproduction of guidelines with frontline users to support practicality and relevance (53)
		Training	Staff frequently make discretionary decisions on access that can be vulnerable to unconscious bias.	The introduction of training specific to teleconsultations for clinical and non-clinical staff (25, 106).
				The explicit acknowledgement of the need for discretion in an imperfect system (4).

## 4 Conclusions

To the best of our knowledge this is the first time anywhere, but certainly in the UK, the complex processes of accessing primary care have been isolated, visualised, and described in the context of the impact of policy. Unpicking the various interlinked components via a Service Blueprint has allowed a more precise description of the impact of various policies and service initiatives on a range of established and novel health service processes and interventions (40, 159). The blueprint we created was based on the largest purposely collected qualitive data set yet to explore access to primary care in the UK, and corroborated with a range of policy and peer-reviewed literature (48, 141, 142, 160). We acknowledge that work has only focussed on the UK and that although comprehensive in the range of health policies discussed primary care organisations do not sit in isolation. There may be broader societal and cultural influences on the way in which patients are able, or prefer to, access care. However, it remains a useful demonstration of how a process visualisation can support commissioners and policymakers understanding of the impact of their decisions on patient and staff experience (158, 161, 162).

Safe and consistent access to general practice is an integral element of the equitable and personalised future of NHSE care provision. However, the growing reliance on remote and digital solutions risks leaving large parts of the population disadvantaged and reinforcing existing health inequalities. This brief has provided yet further evidence of how future policy design would benefit from closer attention to the experiences of patients and front-line providers and we recommend in particular that greater efforts are made to consult marginalized communities.
